# The Indeterminate Domain Protein ROC1 Regulates Chilling Tolerance via Activation of *DREB1B/CBF1* in Rice

**DOI:** 10.3390/ijms17030233

**Published:** 2016-02-25

**Authors:** Mingzhu Dou, Shuai Cheng, Baotian Zhao, Yuanhu Xuan, Minglong Shao

**Affiliations:** 1 College of Life Sciences, Shandong Normal University, Wenhua East Road 88, Jinan 250014, Shandong, China; kadmz@163.com (M.D.); tengda888666@sina.com (B.Z.); 2 Northeast Institute of Geography and Agroecology, Chinese Academy of Sciences, Changchun 130102, China; chengshuai@neigae.ac.cn; 3 University of Chinese Academy of Sciences, Beijing 100049, China; 4 College of Plant Protection, Shenyang Agricultural University, Dongling Road 120, Shenyang 110866, Liaoning, China; 5 School of Pharmaceutical Science, Wenzhou Medical University, Wenzhou 325000, Zhejiang, China

**Keywords:** ROC1, indeterminate domain, cold stress, *CBF1*, rice

## Abstract

Abiotic stress, including salinity, drought and cold, severely affect diverse aspects of plant development and production. Rice is an important crop that does not acclimate to cold; therefore, it is relatively sensitive to low temperature stress. Dehydration-responsive element-binding protein 1s (DREB1s)/C-repeat binding factors (CBFs) are well known for their function in cold tolerance, but the transcriptional regulation of *CBFs* remains elusive, especially in rice. Here, we performed a yeast one-hybrid assay using the promoter of *CBF1*, a cold-induced gene, to isolate transcriptional regulators of *CBF1*. Among the seven candidates identified, an indeterminate domain (IDD) protein named ROC1 (a regulator of *CBF1*) was further analyzed. The *ROC1* transcript was induced by exogenously-treated auxin, while it was not altered by cold or ABA stimuli. ROC1-GFP was localized at the nucleus, and ROC1 showed trans-activation activity in yeast. The electrophoretic mobility shift assay (EMSA) and ChIP analyses revealed that ROC1 directly bound to the promoter of *CBF1*. Furthermore, *ROC1* mutants exhibited chilling-sensitive symptoms and inhibited cold-mediated induction of *CBF1* and *CBF3*, indicating that ROC1 is a positive regulator of cold stress responses. Taken together, this study identified the *CBF1* regulator, and the results are important for rice plant adaptation to chilling stress.

## 1. Introduction

Cold is an abiotic stress that is separated into two parts; one is chilling (<20 °C for many plants), and the other one is freezing (<0 °C) temperature. Cold limits the spatial distribution of plants and adversely affects agricultural quality and productivity [1]. Cold acclimation is a process to increase freezing tolerance by experiencing a low non-freezing temperature before freezing stress [2]. Rice (*Oryza sativa*) is one of the major cereal plants worldwide; therefore, a higher yield potential and yield stability are needed to meet the challenges of increasing demand for rice production [3]. However, world climate changes have become serious problems for many crops when they experience a relatively lower temperature and dehydration [2]. Therefore, isolation of genes involved in the cold stress tolerance response may be an important approach to develop rice plants that can resist low temperature damage. 

One of the strategies by which plants adapt to environmental changes is the rapid reprograming of the transcriptome. Many genes respond to low temperature, including some transcription factors, which are primarily important for understanding cold signaling [1]. Among the transcription factors regulated by cold, transcription activators DREBs1/CBFs have been identified to regulate many cold-responsive genes carrying a specific *cis*-element motif ((DRE; 5’-TACCGACAT-3’)/C-repeat (CRT; 5’-TGGCCGAC-3’) with a common core motif (5’-CCGAC-3’)) in their promoters [4]. In Arabidopsis, *AtCBF1/DREB1B*, *AtCBF2/DERB1C* and *AtCBF3/DREB1A* are rapidly induced by cold stress [5,6]. In rice, *OsDREB1B* and *OsDREB1A* are quickly induced, while *OsDREB1C* is not altered upon cold stress [7]. Further, overexpression of *AtCBF1*, *AtCBF3* and *OsCBF3* activated the transcription of genes carrying the DRE/CRT element in their promoters, even under normal growth conditions in Arabidopsis [4,7,8,9,10]. However, *AtCBF2* negatively regulated freezing tolerance via the negative regulation of *CBF1* and *CBF3* [11]. 

HOS1, ICE1 and MYB15 were identified to directly regulate *CBF/DREB1* expression in Arabidopsis [12,13,14]. The high expression of osmotically-responsive gene 1 (HOS1), which is an RING-type ubiquitin E3 ligase, negatively regulated cold-induced *CBF/DREB1* expression [15]. The inducer of *CBF/DREB1* expression 1 (ICE1) encodes a MYC-like basic helix-loop-helix transcription factor that activates *CBF/DREB1* expression in a cold-dependent manner [16]. MYB15 represses the expression of *CBF/DREB1* via binding of *CBF/DREB1* promoter to regulate freezing tolerance, and it also physically interacts with ICE1, which in turn attenuates MYB15 expression [12]. However, transcriptional regulators that directly regulate *CBFs/DREB1s* transcription have not been reported in rice.

The *indeterminate domain* (*IDD*) genes have been characterized as playing roles in diverse aspects of plant metabolism and development. ID1 regulates the flowering time in maize and rice [17,18]. The *magpie*/*IDD3* (*MAG*) and *jackdaw*/IDD10 (*JKD*) genes determine root fate, whereas *IDD1*/*ENY* (*enhydrous*) and *IDD8* are involved in the regulation of metabolic processes for seed maturation and plant development in *Arabidopsis* [19,20,21]. The *Arabidopsis shoot gravity response 5*/*IDD15* (*SGR5*) gene and rice *Loose Plant Architecture1/IDD14* (*LPA1*) gene are involved in shoot gravitropism [22,23]. We previously demonstrated that IDD10 regulates NH_4_^+^-dependent gene expressions in rice roots [24]. In this study, we identified a novel regulator of the rice *CBF1* gene ROC1 from yeast one-hybrid screening and found that ROC1 encodes an indeterminate domain protein. Further, molecular and biochemical assays revealed that ROC1 directly bound to the promoter of *CBF1*. Loss of function of *ROC1* showed a decrease in cold response, and *roc1* mutant plants were hypersensitive to chilling stress. This study was to isolate and characterize a direct regulator of *CBF1*, and we identified an IDD protein function in chilling tolerance responses in rice.

## 2. Results

### 2.1. Isolation of the Regulators of CBF1

**Figure 1:**
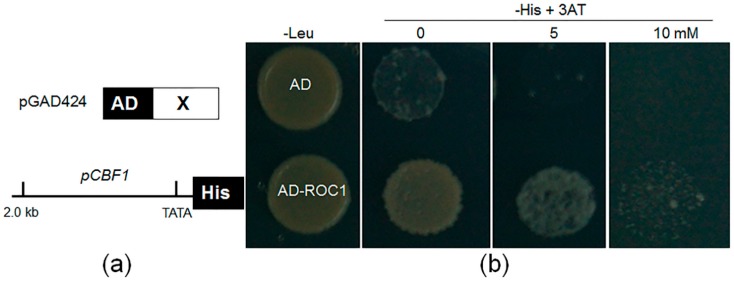
Identification of a regulator of the *CBF1* gene. (**a**) A rice complementary DNA (cDNA) library was generated using a pGAD424 vector in which the coding sequences of the protein of interest were C-terminally fused to the activation domain (AD). A 2.0-kb section of the *CBF1* gene promoter was cloned into the pHISi vector in which His was a reporter gene; (**b**) A yeast one-hybrid assay was performed to analyze the regulator of the *CBF1* gene (ROC1) activation of the *CBF1* promoter. Yeast cells harboring either AD-ROC1 and *pCBF1*-His or AD and *pCBF1*-His were grown on SD media lacking Leu or His and containing the indicated concentrations of 3-amino-1,2,4-triazole (3AT), a competitive inhibitor of HIS3.

*DREB1s/CBFs*, key transcription factors, are induced by cold stress and play a key role in abiotic stress response in plants [2]. Interestingly, rice *CBF1* is different from other *CBF* members that are specifically induced by cold stress [7]. To isolate the putative transcription factors regulating rice *CBF1* expression, we performed a yeast one-hybrid assay using a rice cDNA library and the 2.0-kb *CBF1* promoter to screen for novel proteins binding to the *CBF1* promoter (Figure 1a). The information of the positive interactors is listed in Table S1. The 15 positive clones identified belonged to seven genes, and five clones originated from the same gene (ZOS9-17-C2H2 zinc finger protein, LOC_Os09g38340). Sequence analysis revealed that LOC_Os09g12770 encodes an IDD protein, which we named ROC1 (regulator of *CBF1*). IDD proteins contain a zinc finger domain comprising two C_2_H_2_ and two C_2_HC motifs [17]. The sequence alignment between ROC1 and ID1 showed that they are highly conserved in the ID domain and divergent in the C-terminal region (Figure S1). Two more putative transcription factors (putative zinc finger protein, LOC_Os01g54930; MYB family transcription factor, LOC_Os02g41510) were also isolated with the yeast one-hybrid assay. 

To further analyze the strength of activation of the *CBF1* promoter by ROC1, 3-amino-1,2,4-triazole (3AT), a competitive inhibitor of HIS3, was added to the synthetic defined (SD) medium, and yeast cell growth was monitored. The results showed that the yeast cells expressing AD (activation domain)-ROC1 and *pCBF1*-His were able to grow in the SD media missing histidine and containing 5 mM 3AT, while the empty vector transforming cells failed to grow. However, they also failed to grow in 10 mM 3AT containing SD medium (Figure 1b). These data indicated that ROC1 encodes an ID domain protein and activates the *CBF1* promoter in yeast.

### 2.2. ROC1 Directly Binds to the Promoter of CBF1

**Figure 2:**
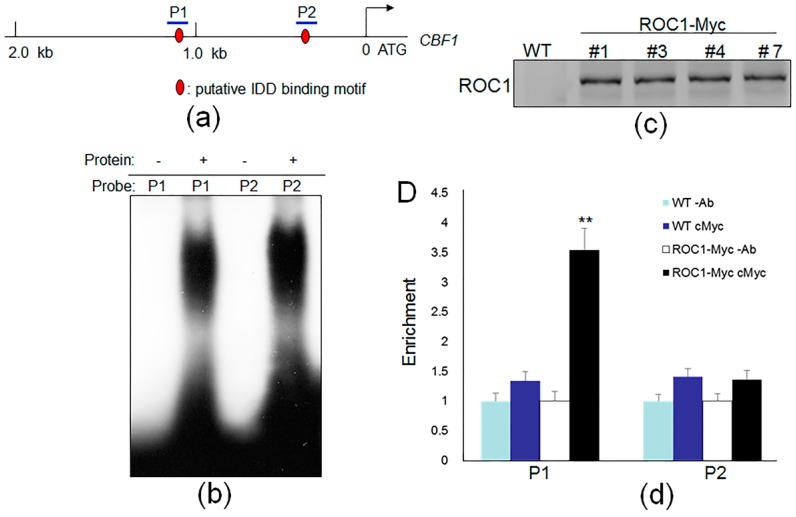
ROC1 directly binds to the promoter of *CBF1*. (**a**) The schematic diagram shows the locations of the putative IDD binding motifs (red oval) in the *CB*F1 promoter and regions (blue line) tested in the electrophoretic mobility shift assay (EMSA) and ChIP assays; (**b**) EMSA was performed to evaluate the ROC1 affinities to each of the putative IDD binding motifs located in the *CBF1* promoter; (**c**) ROC1-Myc expression in *Ubiquitin:ROC1-Myc* transgenic rice plants was analyzed by immunoblotting using an anti-cMyc antibody; (**d**) A CHIP assay was performed by amplifying immunoprecipitated DNA to detect the P1 and P2 regions in the *CBF1*promoter; immunoprecipitated DNA was normalized to input DNA after ChIP-PCR. Data represent the means ± SE (*n* = 3); non-transgenic plants (wild-type) were used as controls. Ab: IgG; cMyc: Myc antibody. ** *p* < 0.01; the *p*-value of the ROC1-Myc sample was calculated with respect to that of the controls.

Because ROC1 is able to activate the *CBF1* promoter in a yeast one-hybrid assay, we further confirmed whether ROC1 binds directly to the *CBF1* promoter. Previous studies reported the motifs that bound the IDD protein in maize (ID1, 5’-TTTGTC^G^/_C_TTTT-3’), Arabidopsis (IDD8, 5’-TTTTGTCC-3’) and rice (IDD10, 5’-TTTGTC^C^/_G_-3’) [21,24,25]. Promoter sequence analysis identified that putative IDD binding motifs were located 1118 to 1125 (P1) and 289 to 295 (P2) bp upstream of the start codon (Figure 2a). To verify the binding affinity of ROC1 to the putative IDD binding motifs, an electrophoretic mobility shift assay (EMSA) was performed using a GST:ROC1 fusion protein. The results showed that ROC1 directly bound to the P1 and P2 sequences (Figure 2b). To analyze ROC1 binding to the promoter of *CBF1 in vivo*, a chromatin immunoprecipitation (ChIP) assay was performed using *ROC1-Myc* transgenic plants. ROC1-Myc lines express *ROC1* cDNA fused to Myc coding sequences, and ROC1-Myc expression was tested by Western blot analysis using an anti-cMyc antibody. The immunoblotting data showed that ROC1-Myc was successfully expressed in the transgenic rice plants, while no visible signal was detected in the non-transgenic plants (Figure 2c). Immunoprecipitates were isolated with IgG and anti-Myc antibodies. Two sets of primers were used to detect P1 and P2 regions using the immunoprecipitates. The ChIP-PCR results indicated that ROC1 is able to bind to the P1 region, but not the P2 region (Figure 2d). Furthermore, competitive electrophoretic mobility shift assays (EMSAs) were performed between P1 and mutated P1 (mP1). The increase of unlabeled P1 content significantly affected the binding between ROC1 and the labeled P1 probe, whereas mP1 slightly interfered with the binding (Figure S2). These results indicated that ROC1 directly binds to the P1 sequences of the *CBF1* promoter.

### 2.3. ROC1 Is Localized in the Nucleus and Shows Trans-Activation Activity in Yeast

**Figure 3:**
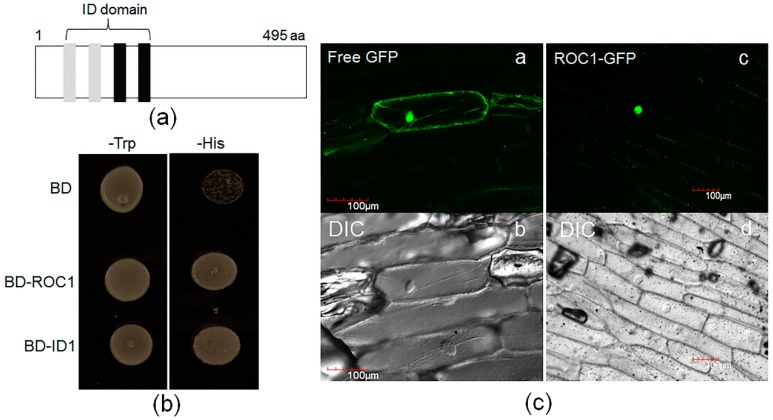
*Trans*-activation and sub-cellular localization of ROC1. (**a**) ROC1 consists of 495 amino acids and encodes an indeterminate domain. Gray and black boxes indicate the C2H2 and C2HC zinc finger motifs, respectively; (**b**) *Trans*-activation activity of ROC1. DNA encoding full-length ROC1 was C-terminally fused to the GAL4 DNA-binding domain (BD) and expressed in yeast cells. Rice ID1 and an empty vector were used as the positive and negative control, respectively. Yeast cells expressing the indicated constructs were grown on SD media lacking Trp or His; (**c**) Localization of free GFP (**a**) and ROC1-GFP (**c**) in onion epidermal cells. GFP indicates the green fluorescence of proteins; (**b**,**d**) DIC indicates the differential interference contrast phase. Bars = 100 µm.

Since IDDs have been reported as transcription factors, the transcriptional activity of ROC1 was analyzed in yeast. In a transcriptional activation assay, GAL4DNA-binding domain (BD) N-terminally fused to the full-length of the *ROC1* open reading frame (ORF) region, which consists of 495 amino acids (Figure 3a). Rice ID1 was used as a positive control [18], while an empty vector was used as a negative control. The ROC1 and ID1 exhibited strong *trans*-activation activity (Figure 3a). To understand the subcellular localization of ROC1, ROC1-GFP was expressed in onion epidermis cells under the control of a non-specific promoter (35S). Free GFP expression was observed in the cytosol and nucleus, while ROC1-GFP was detected in the nucleus (Figure 3b).

### 2.4. ROC1 Mutants Are Sensitive to Cold Stress

**Figure 4:**
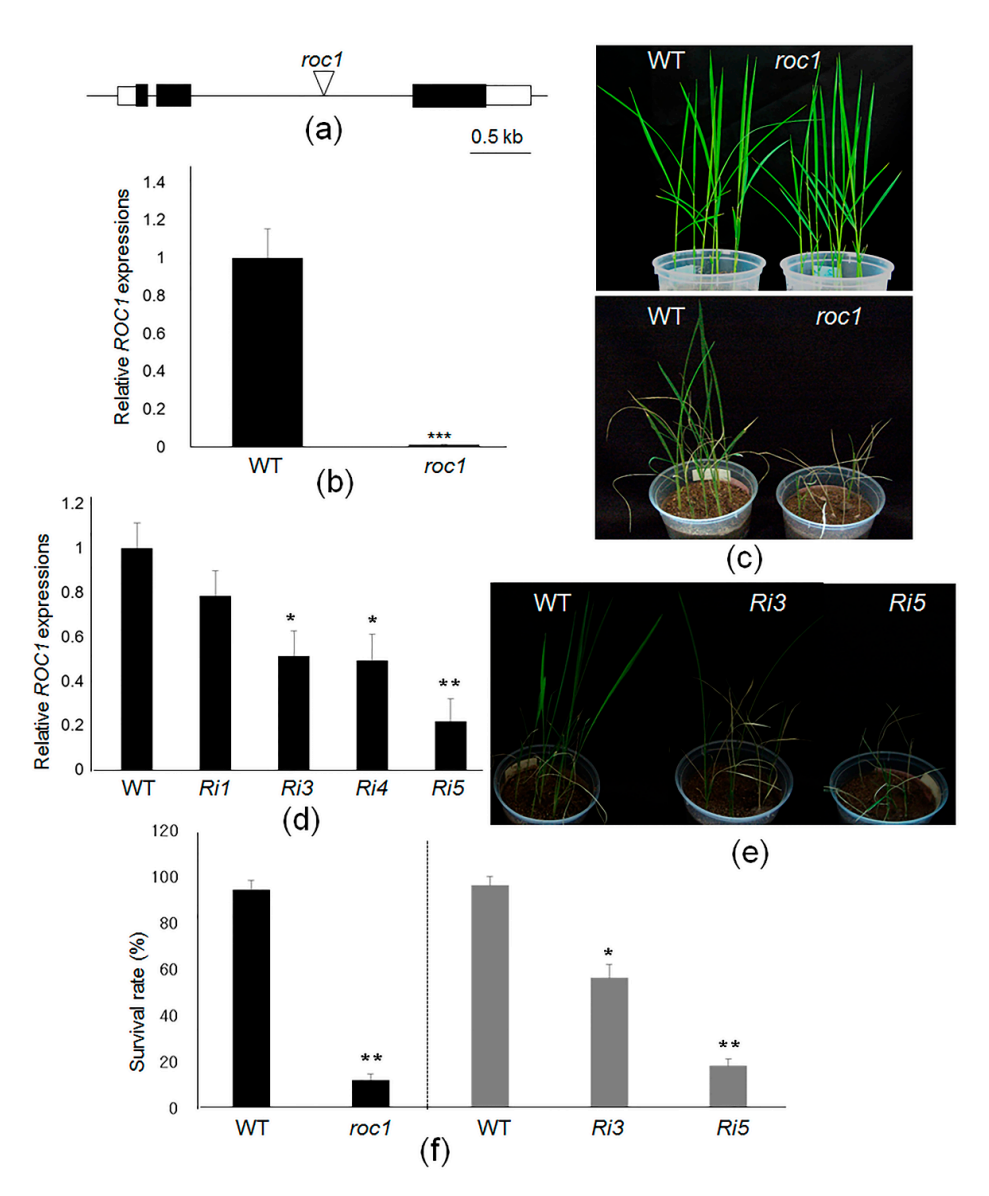
Genomic structure and phenotypic expression of *ROC1* mutants. (**a**) The diagram shows the genomic structure of the *ROC1* T-DNA insertional mutant (*roc1*). Black and white boxes indicate the exons and UTR regions, respectively. The triangle in the second intron indicates the T-DNA insertion sites; (**b**) The *ROC1* expression levels in wild-type (WT) and *ROC1* mutants were analyzed by qRT-PCR. The expression levels were normalized against that of *Ubiquitin* mRNA. A significant difference between the wild-type and mutant was shown (*** *p* < 0.001); (**c**) Fifteen-day-old wild-type and *ROC1* mutant plants grown in a rice growth chamber (28 °C, **upper** side) were further grown in a low temperature growth chamber (4 °C) for four days. The plants were then moved to a 28 °C growth chamber and photographed after 10 days of recovery (**lower** side); (**d**) The *ROC1* expression levels were monitored in four independent wild-type *ROC1* RNAi transgenic plants (*Ri1*, *Ri*3, *Ri*4 and *Ri*5). Significant differences between the wild-type and RNAi lines are shown (* *p* < 0.05, ** *p* < 0.01); (**e**) Wild-type and two RNAi lines (*Ri3* and *Ri5*) grown in a rice growth chamber (28 °C) were further grown in a low temperature growth chamber (4 °C) for four days. The plants were then moved to a 28 °C growth chamber and photographed after 10 days of recovery; (**f**) The survival rates of each line were calculated. Black columns indicate *roc1* and its corresponding wild-type plants while grey columns indicate RNAi lines and their corresponding wild-type plants. Experiments were repeated at least three times, and data represent the mean ± SE (*n* > 10 plants). Significant differences between the wild-type and mutants or RNAi lines are shown (* *p* < 0.05, ** *p* < 0.01).

To detect the role of ROC1 in response to chilling stress, a *ROC1* T-DNA mutant and the corresponding wild-type plants were compared. In this mutant, T-DNA was located in the second intron of *ROC1* gene (Figure 4a). A step before testing the transcript level in a *roc1* mutant, the *ROC1* expression pattern was examined in roots, leaves, shoot apices and flowers tissues. *ROC1* mRNA was expressed in all tissues and was found to be highly expressed in shoot apices (Figure S3). The qRT-PCR results showed that no visible *ROC1* transcript was detected in the *roc1* mutant (Figure 4b). In normal growth conditions in the growth chamber, no visible phenotypes were observed between *roc1* mutants and wild-type plants (Figure 4c). Next, we tested chilling stress responses in wild-type and *roc1* mutants. Fifteen-day-old plants were transferred to a low temperature chamber (4 °C) for four days and moved back to the normal growth chamber (28 °C) to determine the survival rates. After 10 days of recovery, ~94% of wild-type plants were recovered, while ~16% of *roc1* mutants survived (Figure 4c,f). To confirm the *roc1* mutant phenotype, *ROC1* RNAi transgenic plants were produced. More than 10 independent lines were developed, and the expression levels of *ROC1* in four T2 transgenic plants were analyzed by qRT-PCR. The results showed that *ROC1* was suppressed at different levels and that the *Ri5* plant showed strong suppression (~80%; Figure 4d). To verify the phenotypic expression of *roc1* mutants in response to chilling stress, the survival rate was analyzed from *Ri3* and *Ri5* transgenic lines. Wild-type plants have a ~96 survival rate after four days of cold stress. *Ri3* and *Ri5* showed ~56% and ~18% survival rates, respectively, which were correlated with the *ROC1* expression levels (Figure 4e,f). These data indicated that *ROC1* positively regulates chilling tolerance in rice.

### 2.5. Cold-Induction of CBFs Is Inhibited in roc1 Mutants

**Figure 5:**
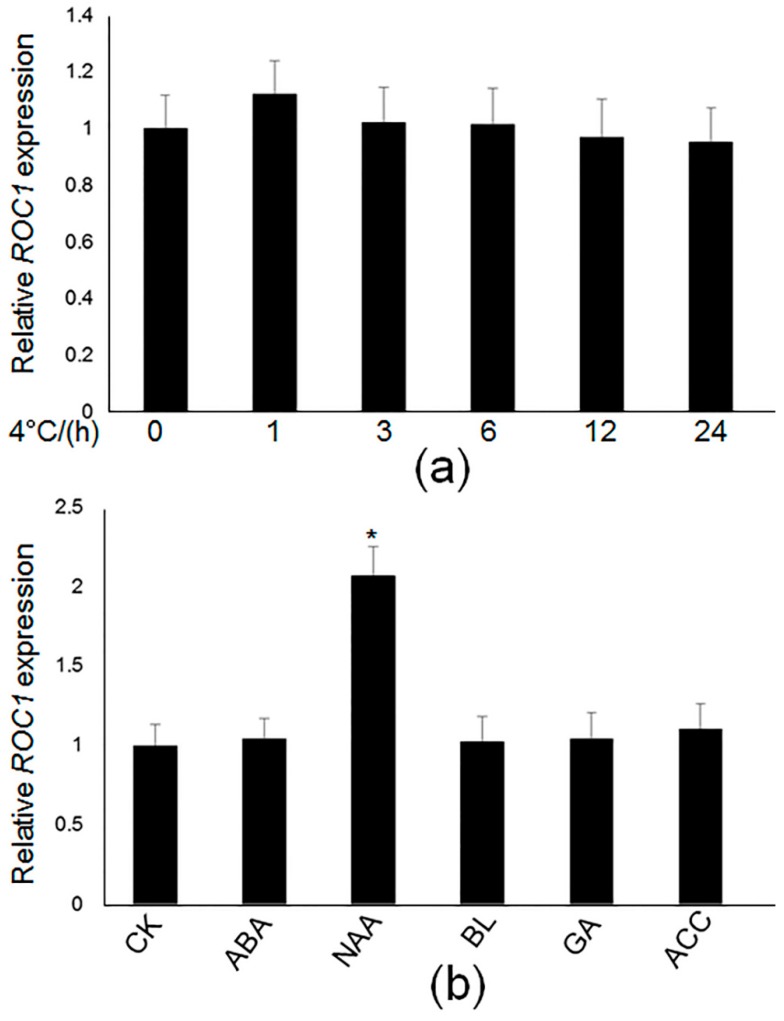
Cold stress and hormone-dependent expression of *ROC1*. (**a**) Fifteen-day-old wild-type seedlings were grown in a normal rice growth chamber (28 °C) and were moved into a low temperature growth chamber (4 °C), and then, the seedlings were sampled after 0, 1, 3, 6, 12 and 24 h. *ROC1* expression levels were monitored with qRT-PCR; (**b**) Hormonal-regulation of *ROC1* transcription. Fifteen-day-old seedlings were treated with 1 μM ABA, 1 μM NAA, 0.1 μM 2,4-epiBL, 1 μM GA and 1 μM ACC for 3 h. qRT-PCR was performed to analyze the expression patterns of *ROC1* upon hormone treatment. The expression levels were normalized against that of *Ubiquitin* mRNA. Significant differences between NAA-treated and -untreated samples (control, CK) are shown (* *p* < 0.05).

Because ROC1 is involved in cold responses, cold-mediated expression of *ROC1* was analyzed. Fifteen-day-old plants were transferred to a low temperature chamber (4 °C), and the seedlings were sampled after 0, 1, 3, 6, 12 and 24 and 48 h of cold treatment. The qRT-PCR results showed that *ROC1* was not altered upon cold treatment (Figure 5a). Phytohormones are known to regulate diverse aspects of plant growth and respond to environmental stresses. Therefore, phytohormone-mediated *ROC1* expression was further examined. After synthetic auxin, NAA (1-naphthaleneacetic acid), BL (brassinolide) GA (gibberellic acid), ethylene precursor, ACC (1amino-cyclopropane-1-carboxylic acid) and ABA (abscisic acid) treatment, *ROC1* was specifically induced by ~1.9-fold by NAA after 3 h of accumulation, but not by other hormones (Figure 5b). 

**Figure 6:**
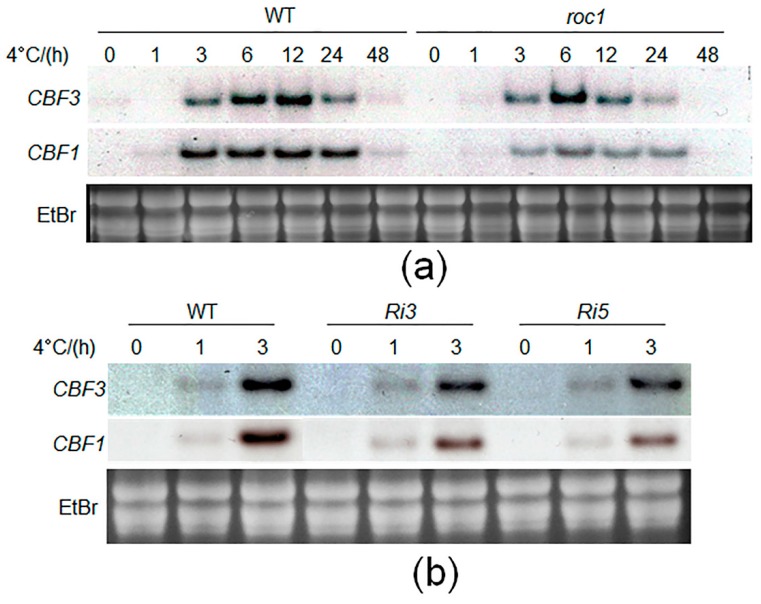
Cold stress-mediated expressions of *CBF1* and *CBF3* in *ROC1* mutants. (**a**) Fifteen-day-old wild-type and *roc1* seedlings were grown in a normal rice growth chamber (28 °C) and were moved into a low temperature growth chamber (4 °C). The seedlings were sampled after 0, 1, 3, 6, 12, 24 and 48 h. The expression levels of *CBF1* and *CBF3* were detected by Northern blot analysis; (**b**) The levels of *CBF1* and *CBF3* were analyzed in wild-type and two *ROC1* RNAi lines (*Ri3* and *Ri5*) after 0, 1 and 3 h of cold treatment. Ethidium bromide (EtBr) staining of rRNA is shown as the loading control.

In the next step, we analyzed the cold-induction of *CBFs* in wild-type and *roc1* mutants. Northern blot analysis shows that *CBF1* is induced for 24 h, while *CBF3* reached the maximum after 12 h of cold treatment (Figure 6a). However, the *CBF1* levels were lower overall at the time points, and *CBF3* reached a maximum after 6 h of cold accumulation in *roc1* knock-out mutants (Figure 6a). Furthermore, the cold-induction of *CBFs* was analyzed in the *ROC1* RNAi lines (*Ri3* and *Ri5*) after 1 and 3 h of cold treatment. The results showed that expression levels of *CBF1* and *CBF3* were lower in two independent RNAi plants after 3 h of cold accumulation (Figure 6b). In sum, these data suggest that *ROC1* is induced by auxin and positively regulates cold-induction of *CBF1* and *CBF3*.

## 3. Discussion

Low temperature is a phenomenon of climate change that affects crop production. To adapt to cold damage, plants trigger the activation of many cold-responsive genes. The CBF family of transcription factors are key regulators during cold acclimation and activate the transcription of genes harboring the DRE/CRT *cis*-element in their promoters [1]. However, cold tolerance requires a cascade of transcriptional regulations. In Arabidopsis, the MYC and MYB types of transcription factors have been reported to directly regulate *CBFs* [12,16]*.* In this study, we performed yeast one-hybrid assays to screen the transcriptional regulators of *CBF1,* which is induced specifically by cold stress [7]. Two zinc finger types and one MYB-like putative transcription factor were selected. Subsequent searches revealed that the putative zinc finger and MYB-like proteins were functionally unknown proteins (Table S1). However, the *MYB* gene (LOC_Os02g41510) was reported for its cold-induction and as the putative paralog of *OsMyb4*, which has a pivotal role in abiotic stress response. Furthermore, OsMyb4 represses its own promoter and Os02g4151 gene promoter, implying that the complex circuit of MYB regulation occurs during cold stress in rice [26]. ZOS9-17 was recognized as an IDD protein and named ROC1, which directly binds to the putative IDD protein binding motifs presented in the promoter of *CBF1*. Analysis of the *roc1* knock-out and knock-down mutants identified that the *ROC1* mutation significantly inhibited the chilling tolerance and cold induction of *CBF1* and *CBF3* (Figure 4 and Figure 6). Because ROC1 positively regulated cold-mediated *CBF3* transcription, the *cis*-elements of the *CBF3* promoter were analyzed. However, no putative IDD binding motifs were observed (data not shown). 

*CBF1-3* are known to act in the ABA-independent pathway to regulate the expression of DRE *cis*-element-containing genes [27]. Hormonal regulation tests for *ROC1* showed that *ROC1* was not altered by ABA treatment, at least at the transcriptional level. Further experiments are required to understand ABA regulation on post-translational modification of ROC1 in the future. However, interestingly, auxin treatment increased *ROC1* levels (Figure 5), and promoter sequence analysis of *ROC1* revealed that auxin responsive elements (AttuRE) are present in the *ROC1* promoter, indicating that ROC1 is involved in auxin signaling. Previous studies reported the connections between auxin biosynthesis or PIN-mediated transport and cold stress [28,29]. Expression tests for *ROC1* identified that *ROC1* does not respond to cold accumulation (Figure 5), but that *ROC1* was required for cold induction of *CBF1* and *CBF3* (Figure 6). ICE1 is a master regulator of *CBFs* in Arabidopsis, the expression of which is sensitive to low temperatures [16]. However, SIZ-mediated SUMOylation at K^393^ of ICE1 significantly inhibited its activity, which in turn repressed the expression of *CBFs* [30]. Previously, we identified that another IDD family member, IDD10, activates NH_4_^+^-mediated gene expressions in rice root, but the IDD10 transcript was unexpectedly repressed upon NH_4_^+^ treatment [24]. More recently, we identified that NH_4_^+^ treatment reduced the phosphorylation level of IDD10 at S^313^ via a phosphoproteomic approach [31], which may affect protein activity. Further biochemical and molecular studies are required to elucidate the complex regulation of ROC1 during low temperature stress.

## 4. Materials and Methods

### 4.1. Mutant Isolation and Plant Growth

The T-DNA insertion line *roc1* (PFG_3A-09378) was obtained from a rice T-DNA database (http://signal.salk.edu/cgi-bin/RiceGE/) [32]. The mutant lines were derived from the Japonica rice cultivar “Dongjin”. Transgenic plants were generated from the Japonica rice cultivar “Nipponbare”.

For testing the low temperature stress response, wild-type, *roc1* mutant and RNAi plants were grown in a rice growth chamber (28 °C, ~30% humidity, 10 h/14 h (light/dark)) for 15 days and transferred to the low Nipponbare temperature chamber (4 °C, ~30% humidity, 10 h/14 h (light/dark)) for 5 days. After 5 days of low temperature treatment, plants were moved to the normal growth chamber (28 °C, ~30% humidity, 10 h/14 h (light/dark)). Fifteen-day-old seedlings grown in a rice growth chamber (28°C, ~30% humidity, 10 h/14 h (light/dark)) were transferred to a low temperature chamber (4°C, ~30% humidity, 10 h/14 h (light/dark)), and the shoots were sampled after 0, 2, 3, 6, 12, 24 and 48 h of cold treatment.

For phytohormone (ABA, NAA, BR, GA and ACC) treatment experiments, plants were grown for 7 days on liquid 0.5X Murashige and Skoog (MS) medium and were transferred to the same medium containing 1 μM NAA (synthetic auxin), 0.1 μM 2,4-epiBL (BR), 1 μM GA, 1 μM ACC and 1 μM ABA for 3 h. Whole seedlings were sampled for RNA extraction. 

### 4.2. Plants Expressing the Plasmid Construction

To generate *ROC1:Myc* transgenic plants, *ROC1* ORF sequences were amplified by primers ROC1 Myc-F and ROC1 Myc-R listed in Table S2 and further cloned into *Hind*III and *BamH*I sites of the PGA1611 binary vector, in which *ROC1* coding sequences were N-terminally fused to *Myc* coding sequences. To generate *ROC1* RNAi plants, 315-bp sequences, including the C-terminal and 3’ UTR regions of the *ROC1* gene, were cloned by primers ROC1 Ri-F and ROC1 Ri-R listed in Table S2. The PCR fragments were digested and cloned into *AscI* and *SwaI* sites in sense and *BamH*I and *Xba*I sites in antisense orientation, respectively, in the pFGC5941 binary vector (ChromDB). 

### 4.3. Yeast One-Hybrid Analysis

To isolate the regulators of *CBF1*, a rice cDNA library using 10-day-old seedlings was established as similar to described previously [33]. A 2.0-kb section of the *CBF1* promoter was cloned using PCR primers pCBF1 F and pCBF1 R listed in Table S2 and cloned into a pHISi vector. Interaction clone screening was performed by a mating method according to the Matchmaker Gold Systems (Clontech) [33]. Among the approximately 1.5 million yeast (cerevisiae) transformants, 15 potential positives clones showed their association with the 2.0-kb *CBF1* promoter. The 15 positive clones selected were further amplified in *Escherichia coli* and sequenced. To verify the interaction between the *CBF1* promoter (2.0 kb) and ROC1, the ORF sequences of *ROC1* were cloned into the pGAD424 vector. The constructed pGAD424-ROC1 or pGAD424 empty vector was transformed into the yeast strain (YM4271), and the growth of yeast cells was monitored on synthetic dropout-Leu or -His together with 3-amino-1,2,4-triazole (3AT), a competitive inhibitor of HIS3.

### 4.4. Subcellular Localization 

*ROC1-GFP* construct was transformed into onion (*Allium cepa*) epidermis cells using the Bio-Rad Helios gene gun system. After bombardment, the onion layers were kept in a growth chamber (22 °C, 24 h dark) for 16 to 20 h. The GFP localization was observed by the Olympus confocal laser scanning microscope [18]. 

### 4.5. Trans-Activation Assays

For testing ROC1 transactivation activity, the Gal4 DNA-binding domain (BD) was N-terminally fused to the *ROC1* and *ID1*ORF regions in a pGBT9 vector and further expressed in the yeast strain PJ69-4A, which contained the *lacZ* and *HIS3* reporter genes [34]. The empty vector (pGBT9) transformed yeast cell was used as the control. Yeast transformants were grown on SD/Trp- and SD/His-plates. The sequences of primers ROC1 BD-F and ROC1 BD-R used for cloning the *ROC1* ORF are listed in Table S2.

### 4.6. RT-PCR Analysis

Total RNA was extracted by using RNeasy Plant Mini Kits [18]. The cDNA was reverse-transcribed from the extracted total RNA using the ReverTra Ace-α- [18]. SYBR Green Master Mix (Bio-Rad) was used for qRT-PCR reaction. A typical qRT-PCR reaction consisted of an initial denaturation at 95 °C for 3 min, followed by 40 cycles with 95 °C for 30 s, 58 °C for 30 s and 72 °C for 30 s, followed by a final extension at 72 °C for 5 min. qRT-PCR products were quantified using the Illumina Research Quantity software (EcoStudy Software v3.0), and the values were normalized against that of *Ubiquitin* mRNA. The sequences of primers (ROC1 RT-F, ROC1 RT-R, Ubiquitin-F and Ubiquitin-R) used for qRT-PCR are listed in Table S2. 

### 4.7. Northern Blot Analysis

One-point-three percent formaldehyde gels were prepared in MOPS (3-(*N*-morpholino) propanesulfonic acid)/EDTA buffer (0.5 M MOPS, pH 7.0; 0.01 M Na_2_EDTA, pH 7.5) for Northern blot analysis. Twenty micrograms of RNA for each sample were heat-denatured in a formaldehyde/formamide solution. The gels were electrophoresed and further washed with 10X SSC for 1 h before being blotted onto a Hybond N^+^ membrane (Amersham Pharmacia Biotech, U.K.). EtBr (ethidium bromide) staining for rRNA was used as a loading control. The membrane and a ^32^P-labeled gene-specific probe were hybridized at 65 °C in Church buffer (1% BSA, 200 µM EDTA, 0.5 M sodium phosphate, 7% SDS). The membranes were autoradiographed using Fuji X-ray film. *CBF1* and *CBF3* fragments were used as probes. The sequences of primers that were used to clone *CBF1* (CBF1 F and CBF1 R) and *CBF3* (CBF3 F and CBF3 R) cDNA fragments are listed in Table S2.

### 4.8. Immunoblot Analysis

The concentrations of the proteins extracted from the seedlings of *ROC1-Myc* transgenic plants were measured with a Bradford protein assay (Bio-Rad, Richmond, CA, USA). Twenty micrograms of ROC1-Myc protein from each line were separated on SDS-PAGE and further transferred to Immobilon-P transfer membranes (MILLIPORE JAPAN, Tokyo, Japan). The membranes were blocked in 1X TBS solution containing 5% skim milk and 0.05% Tween-20 for 1 h, before reaction with an anti-cMyc antibody (Abcam) at 4 °C overnight. An anti-mouse HRP-linked secondary antibody (1:2000, Cell Signaling Technology, Danvers, MA, USA) was used for 1 h. The antigen-antibody complexes were then visualized using an electrochemiluminescence (ECL) kit (GE healthcare, New York, NY, USA).

### 4.9. EMSA

To produce ROC1 recombinant protein, *ROC1* (47 to 212 aa) sequences were sub-cloned into the pGEX5x-1 expression vector. The *pGEX5x-1+ROC1* plasmid was further transformed into *E. coli* (BL21 DE3), and the recombinant GST-ROC1 proteins were harvested after 4 h of 0.5 mM IPTG treatment at 28 °C.

For the EMSA assay, 1 μg of purified GST-ROC1 protein, 40 k cpm (counts per minute) of ^32^P-labeled probe DNA (Amersham, Louisville, KY, USA) and 1 μg of poly (dI-dC) (Sigma, Saint Louis, MO, USA) were reacted in B buffer (25 mM HEPES-KOH (Sigma, Saint Louis, MO, USA), pH 7.5, 100 mM KCl (Sigma, Saint Louis, MO, USA), 0.1 mM EDTA (Sigma, Saint Louis, MO, USA), 10% (*v*/*v*) glycerol (Sigma, Saint Louis, MO, USA) and 1 mM DTT (Dithiothreitol, Sigma, Saint Louis, MO, USA)) at room temperature for 30 min in a total volume of 20 μL. The protein and DNA complex was separated on an 8% polyacrylamide gel (Sigma, Saint Louis, MO, USA) run in 0.5X TBE buffer (Sigma, Saint Louis, MO, USA) [35]. For probe labeling, 20-nucleotide probe DNA was labeled with [γ-^32^P] ATP using T4 polynucleotide kinase (NEB, Ipswich, MA, USA). The sequences of primers that were used for the generation of the EMSA probes (P1 F, P1 R, P2 F, P2 R, mP1 F and mP1 R) are listed in Table S2. 

### 4.10. ChIP Assay

Two grams of young seedlings from non-transgenic and *ROC1-Myc* transgenic plants were used for the ChIP assay. Pre-absorption with a pre-immune serum was performed prior to anti-Myc-mediated immunoprecipitation using a cMyc monoclonal antibody (Abcam, Cambridge, CA, USA). The immunoprecipitates were analyzed by ChIP-PCR. The immunoprecipitates were normalized to the corresponding input DNA for analyzing the relative ratio [35]. The sequences of primers used for ChIP-PCR (P1 ChIP-F, P1 ChIP-R, P2 ChIP-F and P2 ChIP-R) are listed in Table S2.

### 4.11. Statistical Analysis

Statistical analysis was performed by prism 5 (GraphPad, San Diego, CA, USA). Error bars indicate the mean ± SE. Significant differences between the two groups compared were performed by the *t*-test (* p < 0.05; ** p < 0.01).

## 5. Conclusions

Rice is an important cereal plant, but chilling sensitive. To isolate genes responsible for rice cold stress tolerance, transcriptional activators against *CBF1*, a cold specifically-induced gene, were used. The yeast one-hybrid assay and further experiments show that ROC1 directly binds and activates the promoter of *CBF1*. *ROC1* mutants are sensitive to chilling temperature, and cold-induced *CBF1* and *CBF3* are inhibited in its mutants. This study may provide useful information for developing cold-tolerant rice plants in the future. 

## Supplementary Materials

Supplementary materials can be found at http://www.mdpi.com/1422-0067/17/3/233/s1.

## Abbreviations

IDD: Indeterminate domain
CBF: C-repeat binding factor
ROC1: Regulator of CBF1
ABA: Abscisic acid
NAA: 1-Naphthaleneacetic acid
BR: Brassinosteroid
GA: Gibberellic acid
ACC: 1-Amino-cyclopropane-1-carboxylic acid
