# Supplementary material for: The Indeterminate Domain Protein ROC1 Regulates Chilling Tolerance via Activation of DREB1B/CBF1 in Rice
Source: Int J Mol Sci. 2016 Feb 25;17(3):233. doi: 10.3390/ijms17030233 (PMC4813127; doi:10.3390/ijms17030233)
Supplement: Supplementary file 1 [file ijms-17-00233-s001.pdf]

Supplementary Materials: The Indeterminate Domain Protein ROC1 Regulates Chilling Tolerance via Activation of *DREB1B/CBF1* in Rice

Mingzhu Dou, Shuai Cheng, Baotian Zhao, Yuanhu Xuan and Minglong Shao

1. Ethical Statements

Rice transgenic plants were used in this study. The T-DNA insertion mutant roc1 (PFG_3A-09378) was obtained from the SALK rice T-DNA population (http://signal.salk.edu/cgi-bin/RiceGE/, Kyung Hee University, Yongin, Korea). The mutant lines were derived from the Japonica rice cultivar "Dongjin". Transgenic plants, e.g., ROC1 RNAi- and ROC1-Myc- expressing lines, were generated from the Japonica rice cultivar "Nipponbare".

Table S1. Positive interactions from a yeast one-hybrid screen.

Description	Locus No.	Clone Numbers Identified
ATP binding protein	LOC_Os09g30200	2
Putative zinc finger protein	LOC_Os01g54930	2
ZOS9-17 - C2H2 zinc finger protein	LOC_Os09g38340	5
60S ribosomal protein L29-2	LOC_Os05g28750	2
40S ribosomal protein S4	LOC_Os02g01560	1
Expressed protein	LOC_Os08g19050	1
MYB family transcription factor	LOC_Os02g41510	2

Table S2. Primer sequences.

Primers	Sequences
ROC1 Myc-F	GATTCATACAAGCTTATGGCGGCCGCCTCGTCCGCACCCTTC
ROC1 Myc-R	AGATCTGCGGCGGCCATGTTTGCCGGGTCCAGTGAGCCGAC
ROC1 Ri-F	TCTAGAGGCGCGCCATCTCATCATGGGCTCCATG
ROC1 Ri-R	GGATCCATTTAAATAATTCTGATATGATGAAATG
ROC1 BD-F	GAATTCATGGCGGCCGCCTCGTCCGCACCCTT
ROC1 BD-R	GTCGACTCAGTTCATGTTTGCCGGGTCCAG
ROC1 GST-F	GAATTCAAGAAGAAGAGGAACCAGCC
ROC1 GST-R	GTCGACGGGCGGCATGCGCGCGTTC
CBF3 F	ATGTGCGGGATCAAGCAGGAGATG
CBF3 R	CTAGTAGCTCCAGAGTGGGACGTC
CBF1 F	GTGAACCAGAGAGAGTCATCCATG
CBF1 R	TTAGTAGCTCCAGAGCGGCATGTC
P1 F	GGATCCGTCCCTGTTTGATAGCTTG
P1 R	CAAGCTATCAAACAGGGACGGATCC
P2 F	ACCCTGCAGAAACAGCCCCAAAAAC
P2 R	GTTTTTGGGGCTGTTTCTGCAGGGT
mP1 F	GGATCCGTCTTTTTTTGATAGCTTG
mP1 R	CAAGCTATCAAAAAAAGACGGATCC
P1 ChIP-F	CTAGATAGAGTTACAGTC
P1 ChIP-R	CTTCAAACCAAAATTACC
P2 ChIP-F	TACACCTCACCTTACCAC
P2 ChIP-R	CAGCTTCACGGCGTTTTC
pCBF1 F	GAATTCACGAGGAAAATATAACTTTGAC
pCBF1 R	GAGCTCGGATGACTCTCTCTGGTTCACT

Table S2. *Cont.*

Primers	Sequences
ROC1 RT-F	ACCGGGATCAAGAAGCACTACTG
ROC1 RT-R	GATCAAACTGAGAGGCGCCATTG
Ubiquitin-F	GCACAAGCACAAGAAGGTGA
Ubiquitin-R	GCCTGCTGGTTGTAGACGTA

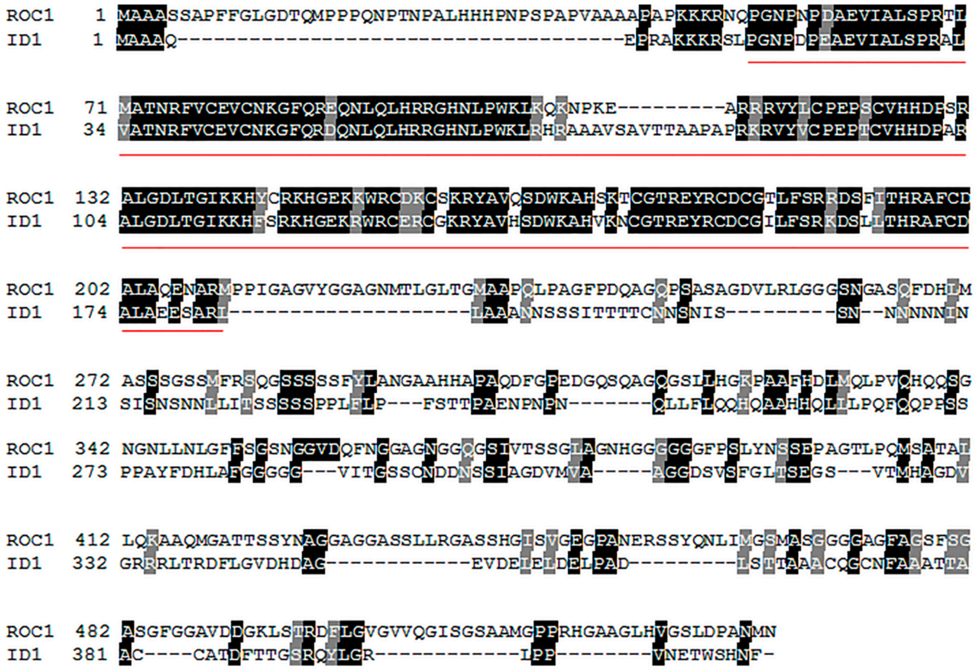

Figure S1. Sequence alignment of ROC1 and ID1. Identical and similar amino acids are shown in black and gray boxes, respectively. Red horizontal bars under the sequences indicate the ID domain.

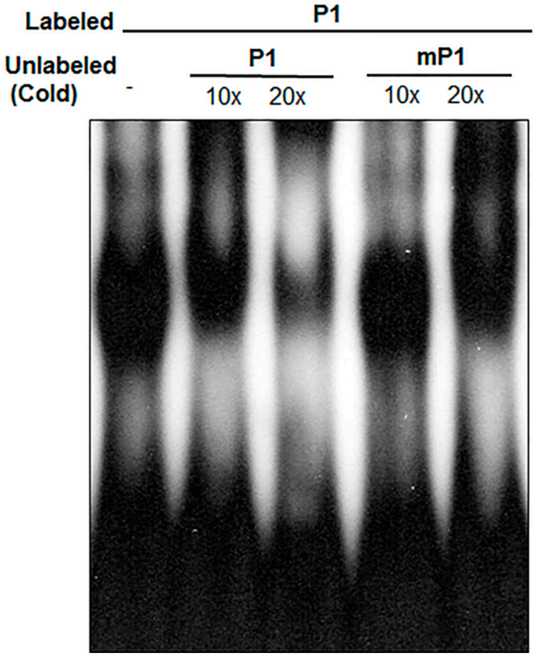

Figure S2. Binding specificity of ROC1 to the P1 sequences. A competition assay was performed with an unlabeled probe to P1 (core sequences: CCTGTTT) and its mutation mP1 (core sequences: TTTTTTT). ROC1 bound to the labeled P1 probe was only significantly affected by increasing amounts of unlabeled P1, whereas mP1 slightly interfered with binding.

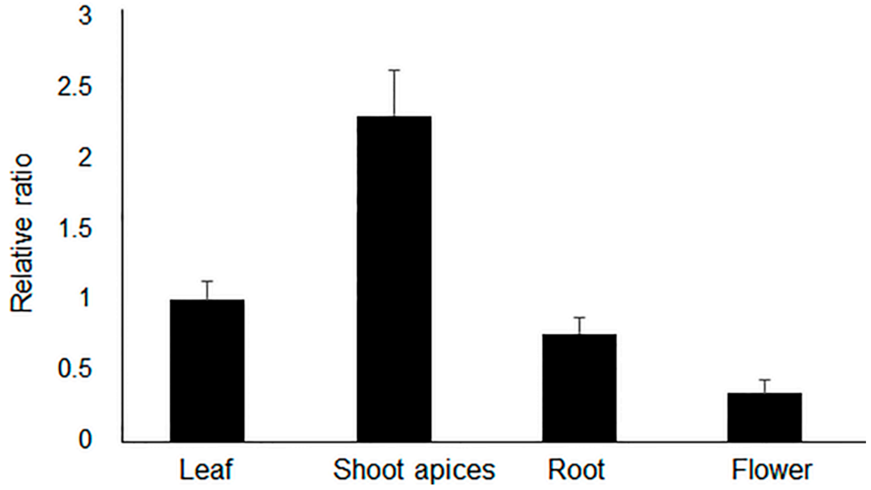

Figure S3. Expression patterns of *ROC1*. qRT-PCR was performed on mRNA from the roots and leaves of one-week-old rice plants, shoot apices of three-week-old plants and flowers of three-month-old plants. The mRNA levels were normalized to that of *Ubiquitin* mRNA. Error bars represent the ± SE of the means from three replicates.
